# Evaluation of a methylation classifier for predicting pre-cancer lesion among women with abnormal results between HPV16/18 and cytology

**DOI:** 10.1186/s13148-020-00849-x

**Published:** 2020-04-21

**Authors:** Yuan-Yuan Gu, Guan-Nan Zhou, Qing Wang, Jing-Xin Ding, Ke-Qin Hua

**Affiliations:** 1 Department of Gynecology, The Obstetrics and Gynecology Hospital of Fudan University, 419 Fang-Xie Road, Shanghai, 200011 People’s Republic of China; 2 Shanghai Key Laboratory of Female Reproductive Endocrine Related Diseases, Shanghai, 200011 China

**Keywords:** Cervical cancer, HSIL, Methylation, S5, Screening, Triage, Classifier

## Abstract

**Background:**

Although HPV testing and cytology detection are successful for cervical screening in China, additional procedures are urgently required to avoid misdiagnosis and overtreatment. In this multicenter study, we collected cervical samples during screening in clinics. A total of 588 women with HPV16/18+ and/or cytology result ≥HSIL+ (high-grade squamous intraepithelial lesion or worse) were referred to colposcopy for pathological diagnosis. Methylation of S5 was quantified by pyrosequencing.

**Results:**

The S5 classifier separates women with ≥HSIL+ from <HSIL with a high area under the curve (AUC) of 0.86 (95% CI 0.840–0.910). The cutoff of 2.85 was conducted in our study. Remarkably, all cancer cases (*n* = 67) were detected by S5. The sensitivity of S5 for “≥HSIL+” was 89.1% (95% CI 86.2–92.4%), and the specificity was 76.6% (95% CI 72.2–78.9%). S5 could reduce unnecessary colposcopy referrals by 74% (95% CI 71.3–78.1%) with virtually no loss of sensitivity for HSIL+, and the follow-up data support the utility of the S5 classifier.

**Conclusion:**

The S5 classifier with high sensitivity and specificity provided increasing diagnostic information for women with HPV16/18+ and/or cytology results and could reduce the numerous unnecessary colposcopy referrals and avoid overtreatment.

## Background

Cervical cancer is a public problem worldwide [1]. It is the fourth most frequent type of tumor among women and the fourth leading cause of cancer death among women [2, 3]. Cervical cancer is primarily caused by human papillomavirus (HPV) infection, and persistent infection with high-risk HPV is a prerequisite for the development of cervical lesions and cancer [4–6]. Not surprisingly, testing for HPV-DNA in cervical cancer screening settings is highly sensitive for the detection of clinically relevant lesions. The disadvantage of HPV-based screening is the modest specificity which results in overtreatment, among young women in particular. However, since most HPV infections will not give rise to (pre)malignant disease, an important concern related to this approach is the increasing unnecessary colposcopy referrals and medical costs [7–9]. At present, reflex cytology detection has been implemented as a routine classifier for cervical cancer screening in the Chinese algorithm. It is widely accepted that cytology is subjective and prior knowledge of HPV presence, as is the case in the setting of primary HPV screening, will likely increase the false-positive rate. While colposcopy screening is successful in screening cervical cancer, it still has limitations, such as subjectivity, interobserver variability, and potential infections. These pressing issues can be addressed by an adequate triage classifier for women with abnormal HPV and cytology results.

An objective triage strategy that could be automated and that incorporated molecular tests combined with HPV detection might be able to solve these problems. DNA methylation plays an imperative role in cancer development, and cancer formation is associated with frequent changes in DNA methylation [10–12]. As a primary form of epigenetic inheritance, DNA methylation has been extensively studied and widely used in tumor classification, early detection, therapy targets, and predictive biomarkers of metastasis and recurrence. The hyper-methylation of CpG (Cytosine-phosphate-Guanine site) islands in the promoter region of tumor suppressor genes, a key mechanism in tumorigenesis, could impede gene transcription and result in a decrease or loss of gene function. Similar to other cancers, epigenetic silencing of tumor suppressor genes by promotor hyper-methylation is a common molecular alteration in cervical cancer [13–15]. Methylation of CpG islands in the tumor suppressor genes prevents the binding of transcription factors to the corresponding DNA response elements, resulting in a decrease in gene transcription, and ultimately, a loss of tumor suppressive function, leading to an uncontrolled cell growth and tumor development. It has been shown that aberrant methylation of some tumor suppressor genes such as PTEN, SFRP1, RASSF1A, DAPK, and RUNX3 occurs frequently in cervical cancer. Therefore, the case that alterations in the DNA methylation of specific genes may be a useful biomarker for early cervical cancer detection. Of more than 100 human methylation biomarker genes detected so far in cervical tissue, close to 20 have been reported in different studies, and approximately 10 have been repeatedly shown to have elevated methylation in cervical cancers and high-grade squamous intraepithelial lesions, most prominently CADM1, EPB41L3, FAM19A4, MAL, miR-124, PAX1, and SOX1 [16, 17].

DNA methylation assays targeting host and/or HPV genes may meet this requirement, as they have been shown to have higher sensitivity and similar specificity to LCT (liquid-based cytology) for identification. S5®(careLYFE) methylation is based on targeting regions of HPV16, HPV18, HPV31, and HPV33 combined with the promoter region of the human tumor suppressor gene EPB41L3 [18]. The PCR-based multiplex assay was followed by quantitative pyrosequencing to measure methylation levels of each assay component. However, the S5®(careLYFE) methylation tests for the triage of cervical cancer and “≥HSIL+” among Chinese women remain elucidated. The S5 assay was developed in a colposcopy study and has been well tested in the UK, Canada, and Mexico [19–22]. As the infection rates of HPV31 and HPV33 in China are low, as well as the lower infection rates of HPV31 and HPV33 in HSIL and cancer, Care Me is a methylation classifier consisting of three regions: EPB41L3, HPV16, and HPV18. It costs a third less than S5, and Care Me is undergoing clinical registration trials in China. EPB41L3 is a human tumor suppressor gene, which is reported to be associated with the progression of cancers.

In this study, the aim was to evaluate the performance of a methylation-specific assay (S5®) comprising five marker regions as a triage test among women with discrepant results in China, assess the sensitivity and specificity of S5 methylation for triage HSIL+, and attempt to find evidence to support S5 as an optimal triage classifier.

## Results

**Fig. 1:**
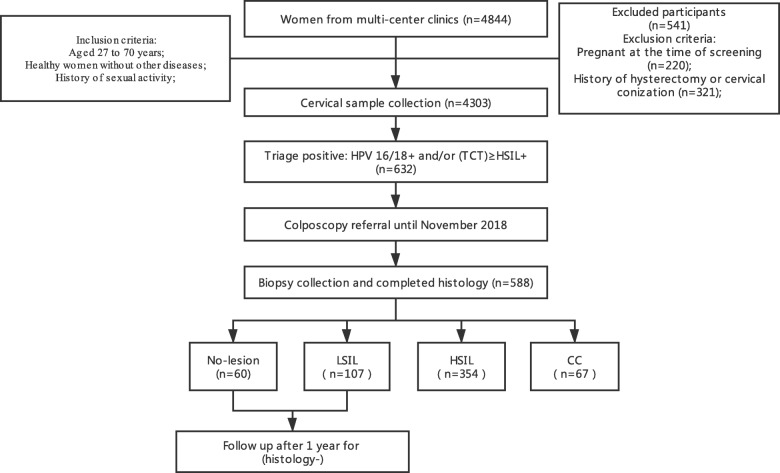
Flowchart of the study showing the numbers of women in each step. Prior to November 2018, 632 of 4303 were called for colposcopy referrals. Then, 588 out of 632 had complete histological results recorded from November 2018 to the study cutoff data on November 1, 2019. These 588 women are considered to represent in the sample frame: 60 no lesion, 107 LSIL, 354 HSIL, and 67 cervical cancer (CC). No lesion, histologically negative without squamous intraepithelial lesion; LSIL, low-grade squamous intraepithelial lesion; HSIL, high-grade squamous intraepithelial lesion; CC, cervical cancer; (TCT)HSIL+, cytological high-grade squamous intraepithelial lesion or worse

The following chart of our study is presented in Fig. 1. The mean age of the final study population (*n* = 588) was 38.8 years (range 27–70 years). Out of the total recruited population, 60 women were diagnosed with no lesion (without cervical lesions), 107 women were diagnosed with LSIL (low-grade squamous intraepithelial lesion), 354 with HSIL, and 67 women with CC (cervical cancer).

As presented in Table 1, the study population’s overall sociodemographic and sexual behavior characteristics did not show statistically significant differences by pathological diagnosis group. Most women were married or had a steady partner (> 85%). Overall, most women had never smoked (> 82%) (Table 1).

**Table 1:** Demographics and sexual behavior characteristics of the female population studied

	Histology				*P* value
	No lesion (*n* = 60)	LSIL (*n* = 107)	HSIL (*n* = 354)	CC (*n* = 67)	
Characteristic	No. (%)	No. (%)	No. (%)	No. (%)	
Age at enrollment					
< 30 years	17 (28.3)	31 (29.0)	79 (22.3)	16 (23.9)	0.080
≥ 30 years	43 (71.7)	76 (31.0)	275 (77.7)	51 (76.1)	0.112
Marital status					
Steady partner*	51 (85.0)	96 (89.7)	326 (92.1)	59 (88.1)	0.061
No steady partner**	9 (15.0)	11 (10.3)	28 (7.9)	8 (11.9)	0.054
Smoker					
No	52 (86.7)	88 (82.2)	311 (87.9)	55 (82.1)	0.064
Yes	8 (13.3)	19 (17.8)	43 (12.1)	12 (17.9)	0.057
Number of sexual partners					
0–1	49 (81.7)	87 (81.3)	286 (80.8)	51 (76.1)	0.093
≥ 2	11 (18.3)	20 (18.7)	68 (19.2)	16 (23.9)	0.088
Time of sexual activity^					
11 years	13 (21.7)	17 (15.9)	51 (14.4)	12 (17.9)	0.073
11–20 years	18 (30.0)	23 (23.4)	101 (28.5)	19 (28.4)	0.092
21–30 years	13 (21.7)	37 (34.6)	98 (27.7)	15 (22.4)	0.054
≥ 30 years	16 (26.6)	30 (28.1)	104 (29.4)	21 (31.3)	0.084

*No lesion* histologically negative without squamous intraepithelial lesion, *LSIL* low-grade squamous intraepithelial lesion, *HSIL* high-grade squamous intraepithelial lesion, *CC* cervical cancer

*Married or with stable partner

**Single women, windows, or divorcees

^Obtained by subtracting the age of onset of sexual life to the age at the time of the study

**Fig. 2:**
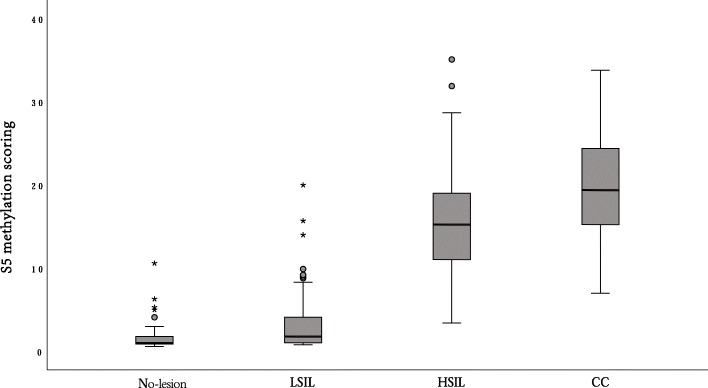
S5 score distributions by histological diagnosis: no lesion, LSIL, HSIL, and cervical cancer (CC). No lesion vs HSIL (*p* < 0.001), no lesion vs CC (*p* < 0.001), LSIL vs HSIL (*p* < 0.001), LSIL vs CC (*p* < 0.001), and HSIL vs CC (*p* < 0.001). The middle line is the median; the Cuzick test for trend was significant (*p* < 0.001). The upper whisker extends to the largest point of the inter-quartile range from the upper quartile. The lower whisker extends to the smallest point of the inter-quartile range from the lower quartile. No lesion, histologically negative without squamous intraepithelial lesion; LSIL, low-grade squamous intraepithelial lesion; HSIL, high-grade squamous intraepithelial lesion; CC, cervical cancer

Figure 2 shows the distribution of the S5 classifier by histological diagnosis. The S5 classifier showed a highly significant increase proportional to the severity of lesions (Cuzick test for trend, *p* < 0.001). Median methylation scoring was 1.1 in histologically negative samples (no lesion), 2.1 in LSIL, 15.2 in HSIL, and 19.7 in cervical cancer (CC). The Mann-Whitney *U* test revealed highly significant differences between the following pairwise comparisons: no lesion vs HSIL (*p* < 0.001), no lesion vs CC (*p* < 0.001), LSIL vs HSIL (*p* < 0.001), LSIL vs CC (*p* < 0.001), and HSIL vs CC (*p* < 0.001).

**Fig. 3:**
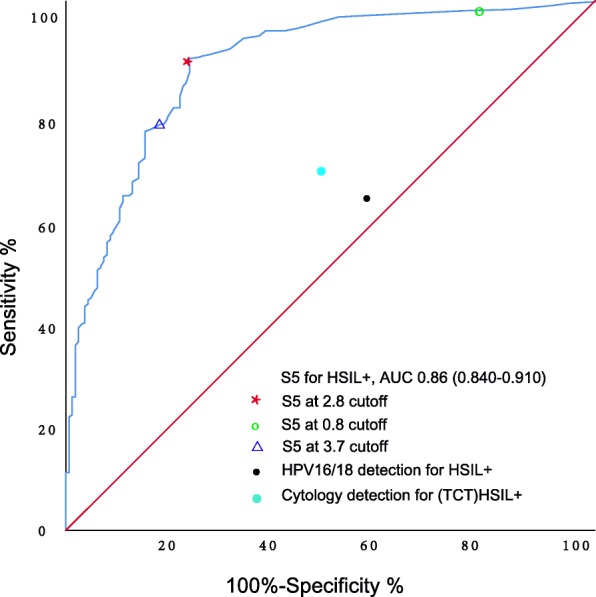
S5 receiver operating characteristic curves for detecting HSIL+. The red star denotes the sensitivity and specificity of S5 at cutoff of 2.85 for HSIL+. The green circle denotes the sensitivity and specificity of S5 at cutoff of 0.8 for HSIL+. The blue triangle denotes the sensitivity and specificity of S5 at cutoff of 3.75 for HSIL+. The cutoff for cytology was ≥ (TCT)HSIL+. HSIL+, high-grade squamous intraepithelial lesion or worse; (TCT)HSIL+, cytological high-grade squamous intraepithelial lesion or worse for TCT result

ROC analysis of the S5 methylation for detecting HSIL+ is shown in Fig. 3, and the area under the curve (AUC) was 0.86 (95% CI 0.840–0.910). A new S5 cutoff value of 2.85 was selected for better discrimination of “HSIL+” lesions from “<HSIL” diagnoses in our already triaged Chinese women with HPV16/18+ and/or abnormal cytology (Fig. 3). This cutoff value reached a relative sensitivity and specificity of 89.1% (95% CI 86.2–92.4%) and 76.6% (95% CI 72.2–78.9%), respectively, for “HSIL+” (Table 3). In addition, we calculated the performance of the S5 methylation classifier with a cutoff value of 3.7, which was previously performing in the detection in Mexico, and a cutoff value of 0.8, which was reported in the UK and Canadian screening populations. We observed that the 3.7 cutoff reached a slight increase in specificity of 80.2% (95% CI 76.1–85.0%) but not significantly, while a worse sensitivity of 77.9% (95% CI 72.5–83.9%) (*p* = 0.015). We observed a better sensitivity for HSIL+ of 98.3% (95% CI 94.8–99.2%) (*p* = 0.004) but a worse specificity of 20.9% (95% CI 17.1–25.8%) (*p* < 0.001) at the 0.8 S5 cutoff value.

**Fig. 4:**
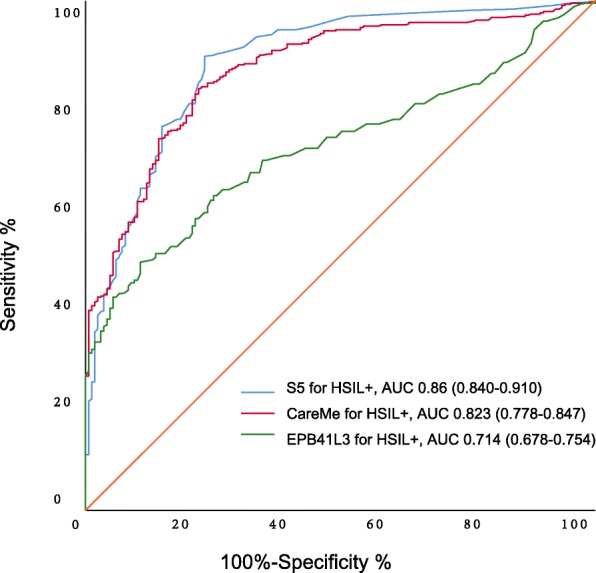
Receiver operating characteristic (ROC) curves of S5 and other existing methylation classifiers for detecting HSIL+. The blue curve denotes the S5 at cutoff of 2.85 for HSIL+. The green curve denotes the EPB41L3 for HSIL+. The blue curve denotes the Care Me for HSIL+. HSIL+, high-grade squamous intraepithelial lesion or worse

ROC analysis of S5 and several existing methylation marker panels for detecting HSIL+ are shown in Fig. 4. The area under the curve (AUC) of S5 with a 2.85 cutoff is 0.860 (95% CI 0.840–0.910), while the AUC of “Care Me” is 0.823 (95% CI 0.778–0.847), and the AUC of EPB41L3 was only 0.714 (95% CI 0.678–0.754). This means that the S5 classifier is better performing in the detection of HSIL+ women compared to “Care Me” and EPB41L3.

We investigated the positivity of different triage tests for no lesion, LSIL, HSIL, and CC histological diagnoses (Table 2). HPV16/18 genotyping was positive in 76.7% (67.7–79.8) of histologically no lesion (histologically negative without squamous intraepithelial lesion) women, while S5 classifier at a cutoff of 2.85 was only 30.8%. HPV16/18 genotyping was positive in 43.0% (39.8–45.7) of histologically LSIL women, positive in 59.9% (54.1–64.2) of histologically HSIL women, and positive in 88.1% (83.2–95.4) of histologically HSIL women. All the 67 cancer cases were identified by S5methylation. However, 11.9% of cancer cases were missed by HPV16/18 detection, and 65.7% of cancers were missed by cytology.

**Table 2:** Positivity of different triage tests for histologically negative (No lesion), LSIL, HSIL, and CC

	No lesion (*n* = 60) % (95% CI)	LSIL (*n* = 107) % (95% CI)	(His)HSIL (*n* = 354) % (95% CI)	CC (*n* = 67) % (95% CI)
HPV16/18+	76.7 (67.7–79.8)	43.0 (39.8–45.7)	59.9 (54.1–64.2)	88.1 (83.2–95.4)
Cytology ≥(TCT)HSIL+	23.3 (19.8–26.4)	57.9 (54.2–60.1)	51.7 (45.9–56.8)	34.3 (28.0–36.1)
S5 at 2.85 cutoff	10.0 (7.6–13.8)	30.8 (27.9–35.0)	87.0 (84.3–89.7)	100
S5 at 3.7 cutoff	8.3 (3.2–13.9)	26.2 (20.2–31.8)	73.7 (69.8–77.6)	100
S5 at 0.8 cutoff	85.0 (77.9–89.4)	75.7 (69.9–78.8)	98.0 (92.4–99.7)	100

*No lesion* histologically negative without squamous intraepithelial lesion, *LSIL* low-grade squamous intraepithelial lesion, (*His*)*HSIL* high-grade squamous intraepithelial lesion, *CC* cervical cancer, (*TCT*)*HSIL+* cytological high-grade squamous intraepithelial lesion or worse

**Fig. 5:**
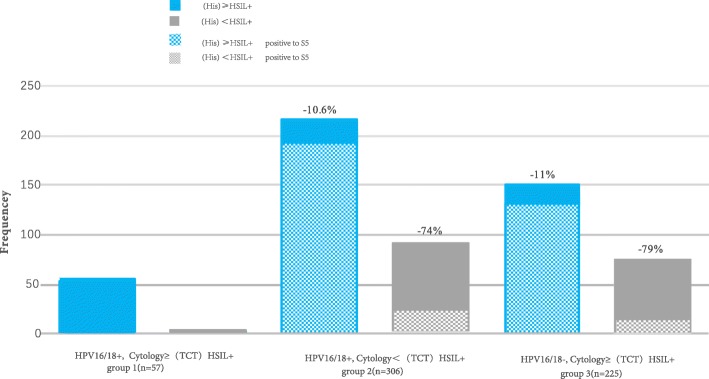
Advantage of conducting S5 classifier as a second triage tool for colposcopy referral for HSIL+ endpoint. We defined three risk groups of women based on the results of HPV16/18 detection and cytology. The group 1 consisted of HPV16/18+ and cytology ≥ (TCT)HSIL+ women. Of all the 57 women in group 1, fifty-six (98.2%) cases were histopathological HSIL+ women; we consider that these women should be referred to colposcopy based on the first triage alone. Group 2 represented a triage discrepancy consisting of HPV16/18+ but cytology < (TCT)HSIL+ women. The use of S5 methylation as a second triage test in this group would have reduced colposcopy referral by 74% for a HSIL+ endpoint (*p* = 0.000). In group 3, women were HPV16/18− but cytology ≥ (TCT)HSIL+, and the use of S5 methylation would have reduced colposcopy referral by 79% for a HSIL+ endpoint (*p* = 0.000). In the both group 2 and group 3, the sensitivity of S5 as the second triage test decreased slightly than in a scenario where all women were referred to colposcopy, but reduce the numerous unnecessary colposcopy referrals by more than 70% to detect HSIL+. The frequency shows the number of women in each group. No lesion, histologically negative without squamous intraepithelial lesion; LSIL, low-grade squamous intraepithelial lesion; HSIL, high-grade squamous intraepithelial lesion; CC, cervical cancer; (TCT)HSIL+, cytological high-grade squamous intraepithelial lesion or worse

As shown in Table 3, the sensitivity of HPV16/18 genotyping for detecting HSIL+ was 64.4% (95% CI 57.8–72.1%), with a specificity of 44.9% (95% CI 40.5–49.1%). Cytology with a cutoff of (TCT (Thinprep cytology test)) HSIL+ (cytological high-grade squamous intraepithelial lesion or worse for TCT result) had a sensitivity of 48.9% (95% CI 42.8–53.2%) and a specificity of 54.4% (95% CI 50.3–58.4%) (here, we define “(TCT)HSIL+” as cytological high-grade squamous intraepithelial lesion or worse for TCT result). In the context of cervical cancer screening in China, a greater specificity would reduce numerous unnecessary colposcopy referrals. We subsequently explored the clinical utility of the S5 methylation classifier as a second or reflex triage test (Fig. 5). We defined three risk groups of women based on the outcomes of HPV16/18 detection and cytology. Group 1 consisted of HPV16/18+ and cytology ≥ (TCT)HSIL+ women. Of all the 57 women in group 1, fifty-six (98.2%) cases were histopathological HSIL+ women. We consider that these women should be referred to colposcopy based on the first triage alone. Group 2 represented a triage discrepancy consisting of HPV16/18+ but cytology < (TCT)HSIL+ women. In our study, these women were also referred to colposcopy, but in retrospect, of all the 306 women in group 2, 91(29.7%) cases were histopathological <HSIL+ women, who were considered to be unnecessarily referred. The use of S5 methylation as a second triage test in this group would have reduced colposcopy referral by 74% for an HSIL+ endpoint (*p* = 0.000). In group 3, women were HPV16/18− but cytology ≥ (TCT)HSIL+, and all these women in our study were referred to colposcopy. Of all the 225 women, 75 (33.3%) cases were histopathological < HSIL+ women, who were considered to be unnecessarily referred. Again, the use of S5 methylation would have reduced colposcopy referral by 79% for an HSIL+ endpoint (*p* = 0.000). In both group 2 and group 3, the sensitivity of S5 as the second triage test decreased slightly compared with in a scenario where all women were referred to colposcopy (group 2, 215/306 vs 192/306; *p* = 0.059; group 3, 150/225 vs 132/225; *p* = 0.079), but the difference was not statistically significant (Fig. 5). This means that conducting S5 methylation as a second triage classifier would reduce numerous unnecessary colposcopy referrals by more than 70% while maintaining a similar sensitivity to detect HSIL+ as the triage based on a combination of HPV16/18 and cytology.

**Table 3:** Performance of HPV16/18 genotyping, cytology, and S5 methylation for detecting (His)HSIL+

	Sensitivity % (95% CI)	Specificity % (95% CI)	PPV % (95% CI)	NPV % (95% CI)
HPV16/18+	64.4 (57.8–72.1)	44.9 (40.5–49.1)	74.6 (71.3–76.8)	33.3 (29.3–38.2)
Cytology ≥(TCT)HSIL+	48.9 (42.8–53.2)	54.4 (50.3–58.4)	73.1 (70.5–75.9)	29.7 (25.4–34.8)
S5 cutoff 2.85	89.1 (86.2–92.4)	76.6 (72.2–78.9)	90.6 (87.2–93.1)	73.5 (69.6–75.7)
S5 cutoff 0.8	98.3 (94.8–99.2)	20.9 (17.1–25.8)	75.8 (70.0–81.4)	85.7 (80.7–89.3)
S5 cutoff 3.7	77.9 (72.5–83.9)	80.2 (76.1–85.0)	90.8 (86.6–94.3)	59.0 (55.9–63.7)

(*His*)*HSIL+* histological high-grade squamous intraepithelial lesion or worse, *PPV* positive predictive value, *NPV* negative predictive value

We followed up the women with histopathological <(His)HSIL+(high-grade squamous intraepithelial lesion for histological diagnosis-positive) 1 year after the colposcopy, and we defined the HPV16/18 negative as regression (when compared with the HPV16/18 positive 1 year before), also we defined TCT<(TCT)HSIL+ as regression (when compared with the TCT ≥ (TCT)HSIL+ 1 year before) (Table 4). Of all the women with S5 scores < 2.85, 40 (58.8%) of 68 HPV16/18+ women regressed to HPV16/18 negative after colposcopy within 1 year, while of those with S5 scores ≥ 2.85, only 1 (4.6%) of 24 cases regressed. Of all the women with S5 scores < 2.85, 42 (71.7%) of 60 women with cytology ≥ (TCT)HSIL+ regressed after colposcopy within 1 year, while of those with S5 scores ≥ 2.85, only 2 (12.5%) of 16 cases regressed.

**Table 4:** The follow-up data of women with histology < (His)HSIL+ according S5 scoring

	S5 scoring < 2.85	S5 scoring ≥ 2.85
HPV16/18 + (*n*)	68	24
HPV16/18 − (1 year later) (*n*)	40	1
Regression rate (%)	58.8	4.6
Cytology≥(TCT)HSIL+ (*n*)	60	16
Cytology<(TCT)HSIL+(1 year later) (*n*)	42	2
Regression rate (%)	71.7	12.5

As the descripted in the title of Table 4, “histology < (His)HSIL+” was diagnosed based on the colposcopy (until 2018 November). One year later (in 2019 November), we followed up women who were diagnosed as “histology < (His)HSIL+.” As these women were referred to colposcopy for HPV16/18+ or cytology ≥ (TCT)HSIL+, we analyzed the HPV16/18 genotyping and cytology in 2019 November, and we define the “regression” as cytology or HPV16/18 becoming negative 1 year later.

(*His*)*HSIL*+ histological high-grade squamous intraepithelial lesion or worse, (*TCT*)*HSIL*+ cytological high-grade squamous intraepithelial lesion or worse.

## Discussion

This study investigated the diagnostic performance of the S5 methylation assay for women already triaged by cytology or HPV16/18 testing and evaluated its utility for potentially decreasing unnecessary colposcopy in the current Chinese algorithm. We also followed up women with “no lesion” 1 year later after colposcopy as corroborative evidence to support the results. These results imply that the S5 could be a potential classifier for cervical cancer and pre-cancer lesion screening.

A great number of studies indicate that persistent hrHPV (high-risk human papillomavirus) infection together with abnormal epigenetic events might lead to cervical pre-cancer lesions and even cervical cancer. When host cells were persistently infected by hrHPV, reactive cellular changes will occur as increased methylation and other epigenetic changes. DNA methylation is detectable for its measurable CpG island signatures and is stably retained during mitotic cell division process [23]. Thus, we explored the clinical utility of the S5 DNA methylation classifier as a triage test in our study of women with abnormal cytology and/or HPV16/18 infections.

As demonstrated in Table 2, S5 methylation assay was able to detect all cancer cases and 89.1% of HSIL+ cases in our study. In comparison, there were still 11.9% of cancer cases missed by HPV16/18 detection. What is more, there were 76.7% histologically no lesion women, and histologically LSIL were referred to colposcopy which might be unnecessary. Meanwhile, 65.7% (44/67) cancer cases were missed by cytology. Of these missed women, 16 cases were cervical squamous carcinomas, 11 cases were cervical adenocarcinomas, 12 were cervical adeno-squamous carcinomas, 3 were cervical neuroendocrine carcinomas, and 2 were cervical clear cell carcinomas. The cytology positive percentage for cervical cancer is lower than normally observed and reported. It is widely accepted that the unsatisfactory results of cytology might result from the experience of observers, the insensitivity to atypical histology, unsatisfactory sampling, and so on. Meanwhile, as this is a multi-center study, all the specimens were collected from each clinic and delivered to the same laboratory. Most of the missed cases in cytology were from the other 2 hospitals (although reviewed by the same cytotechnologists), while the results of HPV and methylation from 3 hospitals were similar. It is highly likely that the transportation influenced the morphology of the cytology slides and misguided the judgements of cytotechnologists. While the HPV and methylation based on the molecular analysis through digital instruments instead of observers, the results in HPV and methylation from 3 hospitals are similar. What is more, the colposcopies were all conducted in the same hospital, named the Obstetrics and Gynecology Hospital of Fudan University, which can guarantee accuracy of colposcopy and histological diagnosis.

Several methylation markers and commercially available methylations kits have been reported to triage the cervical cancer and pre-cancer lesions. The sensitivity of the PAX1 methylation test [24] for the detection of CIN3+ showed a sensitivity of 64% and a specificity of 91%. The sensitivity of the SOX1 methylation test [24] for the detection of CIN3+ showed a sensitivity of 71% and a specificity of 77%. The sensitivity of the POU4F3 methylation test [25] for detecting CIN3+ was 74%, and the specificity was 89%. A methylation study based on MAL and CADM1 showed [26] a sensitivity of 68% and a specificity of 75% for detecting CIN2+. A study based on the combination of EPB41L3, JAM3, TERT, and C13ORF18 showed [27] the sensitivity of 65% and a specificity of 79% for detecting CIN2+. A methylation study based on the 5′ regions of the genes DLX1, ITGA4, RXFP3, SOX17, and ZNF671 showed [28] the sensitivity of 43.1% and a specificity of 88.7% for detecting CIN2/3. In a self-sampling study [29], DNA methylation testing for MAL and miR-124 showed a sensitivity of 70.5% for detecting CIN2+. Also, some methylation kits were tested, such as the QIAsure [10] (based on FAM19A4 and has-mir124-2) and GynTect assay [30] (based on ASTN1, DLX1, ITGA4, RXFP3, SOX17, and ZNF671). The QIAsure methylation based on FAM19A4 and has-mir124-2 showed a sensitivity of 70.5% and a specificity of 67.8% for detecting HSIL+. And the GynTect methylation based on ASTN1, DLX1, ITGA4, RXFP3, SOX17, and ZNF671 showed a sensitivity of 64.8% and a specificity of 94.6% for detecting HSIL+. Since these studies were conducted within different population and set different endpoint, the comparison of different methylation tests needs further normalization. Nevertheless, S5 with high sensitivity and high specificity would provide additional information for women with discrepant results of HPV16/18 and cytology.

Another encouraging finding of our study is re-analysis of the diagnostic utility of S5 reported in three earlier studies, a large screening study in the UK, a nested case-control triage study in Mexico [18], and a randomized control trial in Canada [21]. In the hrHPV-based screening study from the UK, using a cutoff of 0.8 for S5, the AUC of the 0.8 cutoff was 0.78 (95% CI 0.69–0.88) for HSIL+ but had a lower specificity of 20.9%. In the nested case-control triage study in Mexico, using a cutoff of 3.7 for S5, the AUC obtained for HSIL+ was only 0.75 (95% CI 0.69–0.8), lower than the AUC (0.86) of 2.85 cutoff.

In previous studies of S5 performance [20], the sensitivity for cancer at the suggested triage cutoff of 0.8 was 100%. In our study, the results showed that even at a cutoff of 2.85, all cancers were detected. Similar data have also been shown in other studies, which indicate few if any cancers would be missed when employing DNA methylation signatures for triage, even with a rather high S5 cutoff of 2.85 or perhaps higher.

It is widely accepted that most cervical pre-cancer lesions result from hrHPV infections and most of them regress and only a small number of them (< 5%) progress toward cervical cancer [31]. To decrease costs and to avoid a large amount of unnecessary treatment, it is of great significance to distinguish women who are most likely to develop truly pre-cancerous high-grade SIL from women with “HSIL” but will never progress toward cervical cancer or at least not over several decades. Previous studies have proposed classifiers based on the methylation of HPV types and/or genes. Also, S5 performed better than the single EPB41L3 methylation marker and “Care Me” including EPB41L3, HPV16, and HPV18 which derived from S5 assay.

Based on the results in our study, we believe that a combined triage strategy with remarkable specificity and a sensitivity of more than 80% for (His)HSIL+ is feasible and cost-effective in China and many low- and middle-income countries (LMICs). On the one hand, it is not crucial to detect all pre-cancer lesions in the first screening because most of pre-cancer lesions regress or progress slowly. However, cancers are not reversible, and S5 classifier detected all cancer cases in our study and three other previous studies [18–20]. These results indicate that S5 is safe for detecting all cancer cases and 89.1% of (His)HSIL+ cases. On the other hand, S5 classifier would reduce unnecessary colposcopy referrals. A pressing priority in China and indeed in most LMIC is to not overwhelm colposcopy clinics. A workload increase in these clinics could lead to increasingly poor outcomes and potentially to increase in cancer due to hurried and inadequate assessment of women, who may be given false-negative diagnoses or who may be lost to follow-up. Thus, our results support a proposal to set up an enhanced risk scoring algorithm that combines methylation of target genes with current cytology and HPV16/18 triage testing options for hrHPV screen-positive women.

Excessive burdening of clinical services and overtreatment of women are negative features of medical practice today [32, 33]. It might take more than 1 month to make an appointment for a colposcopy in many Chinese hospitals and even longer in other parts of the world. Moreover, overtreatment is still a major issue due to the side effects of invasive therapy such as perinatal loss, preterm birth, bleeding, long-term absence from work, and mental stress [33, 34]. In China, many women with “LSIL” choose invasive treatment such as LEEP or laser treatment in fear of the disease progression. The procedure was unnecessary for many studies reporting that spontaneous regression of LSIL/HSIL lesions to normal histopathology was shown to be as high as 30% within 36 weeks without any treatment, and in another study, the regression rate even reached 39% within 16 weeks [1, 9].

Our results are applicable to methylation triage of women who are discrepantly positive for HPV16/18 and cytology (which means HPV16/18+ with cytology <(TCT)HSIL+ or HPV16/18− with cytology ≥ (TCT)HSIL+); however, we were not able to assess whether S5 could be used for triage in all hrHPV-positive women. Therefore, according to the follow-up results, S5 test may also be beneficial for women who had a normal colposcopy result. In this scenario, a negative methylation result (S5 < 2.85) would provide reassurance against potential missed cervical pre-cancer lesion, especially for women who might be lose to follow-up. A positive methylation result (S5 ≥ 2.85) could be carefully evaluated in follow-up visits to document quantitative changes in methylation consistent with the clearance or progression of disease. This is in line with the results of previous studies that have observed that DNA methylation can predict the persistence of HPV infection of different viral types [6, 10, 35]. However, it needs to be validated in different settings of LMICs to estimate the clinical utility in populations with a similar background of income and disease prevention strategies to estimate the risk of cervical cancer.

Based on the practice of our study, the cost of an S5 test is not higher than that of a routine HPV screening and reflex genotyping, and it could be balanced against the expected 30 to 50% reduction in colposcopy referral costs. If confirmed by more studies, this observation may promote rapid reduction in cervical cancer in LMICs, which has been particularly poorly severed by cytology screening over the past several decades. Although the HPV vaccine program would control HPV infections in many developed countries, expected large-scale cervical cancer incidence reductions are still many decades away, and it is prudent to take more preventative actions.

Our study has some important strengths. This is the first screening multi-triage study of cervical cancer in China. All primary clinical and laboratory procedures (screening, triage, colposcopy, and histopathological assessments) were performed completely within China. We selected all available cases based on strict adherence to the specific principles strictly. The accuracy of the colposcopy evaluation in this study was very high. At least one biopsy was taken from each quadrant of the cervix in all women to reduce verification bias. In addition, the histopathological evaluation and diagnostic confirmation of the biopsies and/or endocervical samples of all women included in our study were reviewed by a panel of pathologists. Moreover, all methylation measurements were performed by blinded laboratory staff, with the aim of avoiding differential information bias.

## Conclusion

The S5 methylation classifier with high analytical sensitivity, specificity, and positive predictive value may play an important role as a triage tool for colposcopy referral in Chinese women with HPV16/18+ and/or abnormal cytology (≥(TCT)HSIL+) results. S5 methylation can reduce unnecessary colposcopy referrals by 70% as compared to conventional screening by HPV16/18 and cytology while detecting all cancer cases. Moreover, S5 might be a potential classifier in LMICs for reducing costs and encouraging doctors to focus on the women at real risk of cervical cancer.

## Methods

### Study population

This study population included women, aged from 27 to 70 years, who visited gynecology clinics in four hospitals for routine cervical cancer screening between December 2017 and November 2018. We performed a cross-sectional analysis in a subset of women with HPV16/18+ and/or abnormal cytology (≥(TCT)HSIL+) results in this study, all of whom underwent colposcopy (Fig. 1). The aim of this study was to evaluate the performance of different triage tests for women with HPV positive and/or abnormal cytology results. Women who were pregnant at the time of recruitment or had a prior hysterectomy were excluded.

This study was approved by the Obstetrics and Gynecology Hospital of Fudan University Institutional Review Board, and the study was conducted in accordance with the Declaration of Helsinki and the International Conference on Harmonization of Good Clinical Practice. Oral and written informed consents were obtained from the patients or their guardians before the study, and those who agreed were administered a survey to collect demographic data.

### Study procedure

Each participant underwent a gynecological examination to collect cervical samples at the screening visit in multi-center clinics, and we conducted the HPV16/18 detection and cytology and S5 methylation assays. In the current Chinese algorithm, women with HPV16/18+ and/or cytology results ≥(TCT)HSIL+ are referred to colposcopy. According to “The Lower Anogenital Squamous Terminology Standardization Project for HPV-associated Lesions” (LAST), we categorized histopathological results as “no lesion,” “LSIL,” “HSIL,” and “CC.” Based on the histopathological diagnoses as the “gold standard,” we compared the diagnostic performance of S5, HPV16/18, cytology, and other existing methylation marker panels. One year after colposcopy evaluation, we followed up women with < (His)HSIL+ to validate the utility of S5 to reduce unnecessary colposcopy referrals (here, we define “(His)HSIL+” as high-grade squamous intraepithelial lesion for histological diagnosis).

### Collection and shipment of cervical sample

All the cervical samples were obtained from the screening visit in multi-center clinics. During the visit of each participant, one cervical sample was collected in a Sure-Path vial and used for the cytology procedure, and another cervical sample was collected for HPV detection and methylation detection, using the HC2 DNA Collection Device (QIAGEN, Gaithersburg, Maryland). Two samples were collected in random sequences in order to minimize bias. All the samples were sent to the laboratory on dry ice.

### Cytology detection

All cytological samples were collected immediately by using a Cervi-Brush® bush (Rovers® Medical Devices, North Brabant, Netherlands). The vials used for the TCT test were stored at 2 °C to 8 °C upon arrival in the central cytology laboratory of Gy&Ob Hospital of Fudan University in Shanghai, China. Liquid-based cytology slides were reviewed by 2 independent cytotechnologists who were blind to their counterpart’s results. If both cytopathologists reported an abnormality, the worst result was established as the final diagnosis. Otherwise, a senior cytopathologist determined the final diagnosis. The cytopathologist also evaluated 10% of the slides with normal findings and all slides with ASC-US findings or worse. All the cytopathologists were blinded to the HPV infection status. Herein, we categorized all the cytology results as “NILM” (no intraepithelial lesion or malignancy), “LSIL,” “HSIL,” and “CC.” The cytology result “ASCUS” (atypical squamous cells of undetermined significance) was grouped as “LSIL,” and “ASC-H” (atypical squamous cells cannot exclude a high-grade squamous intraepithelial lesion) was grouped as “HSIL.”

### HPV detection

Vials for HPV DNA testing were transported to the HPV laboratory of Gy&Ob Hospital of Fudan University in Shanghai, China, and stored at 2 °C to 8 °C until testing could take place. Samples were tested for hrHPV by using the Cobas® 4800HPV test (Roche Molecular systems), a qualitative in vitro assay that identifies a pooled result for HPV16, HPV18, and other 12 other high-risk HPV types, including HPV31, 33, 35, 39, 45, 51, 52, 56, 58, 59, 66, and 68.

### Methylation assays

Vials for methylation testing were transported to the Shanghai Key Laboratory of Female Reproductive Endocrine Related Diseases in Shanghai, China, and stored at 2 °C to 8 °C until testing could take place. Genomic DNA was extracted using 100 μL of suspensions with the QIAamp DNA Micro Kit (Qiagen, Hilden, Germany). DNA was quantified by UV absorption. A total of 250 ng of DNA was used in the bisulfite treatment where un-methylated cytosines were converted to uracil with the EpiTect Fast Bisulfite Kit (Qiagen, Hilden, Germany) according to the manufacturer’s instructions. Converted DNA was eluted in 15 μL Buffer EB twice, and the eluant was combined for the next steps.

Methylation assays were based on pyrosequencing with a single PCR amplicon of less than 300 bp as previously described with slight modification. Primers (for PCR and pyrosequencing) were designed using the PyroMark Assay Design software 2.0.1.15 (Qiagen). PCR for each gene was performed in a total volume of 25 μL containing 2–4 μL of converted DNA, 12.5 μL of 2× PyroMark PCR master mix (Qiagen) and optimized concentrations of primers and magnesium chloride. Generally, reactions were initiated at 95 °C for 15 min, followed by 45–50 cycles of 94 °C for 30 s, 30 s at the annealing temperature, and 30 s at 72 °C, and then a final extension for 10 min at 72 °C was applied. Quantitative methylation analysis of target CpG sites was conducted on a PyroMark Q48 Autoprep system (Qiagen) with recommended protocols and conditions: 10 μL of PCR product was pyrosequenced with 4 μM primers. Raw data were analyzed by the software. A non-CpG cytosine in the pyrosequencing region was taken for each assay as the control for total bisulfite conversion. Positive and non-template negative controls were tested in each run in parallel.

### Colposcopy evaluation

Endocervical curettage was performed for all women referred for colposcopy evaluation, and the colposcopist conducted one biopsy from the most suspected abnormal zone of each quadrant during the procedure. Evaluation of all the histological samples was conducted by a standardized group of pathologists. If both pathologists agreed in their diagnosis, the result was the final diagnosis. Otherwise, the expert pathologists determined the final diagnosis. According to “The Lower Anogenital Squamous Terminology Standardization Project for HPV-associated Lesions” (LAST), we categorized histopathological results as “no lesion,” “LSIL,” “HSIL,” and “CC.”

### Data analysis

Demographic and sexual behavior of participants were summarized as the means or proportions. Differences across all variables between no lesion cases, LSIL cases, HSIL cases, and CC cases were tested by use of a one-way ANOVA test. Our primary aim was to evaluate the clinical diagnostic performance of the S5 methylation classifier. The standardized equation for S5 scoring was used to calculate the average methylation values of the five target regions as follows:

S5 = EPB41L3 × (30.9) + HPV16L1.3 × (13.7) + HPV16L2 × (4.3) + HPV18L2 × (8.4) + HPV31L1 × (22.4) + HPV33L2 × (20.3)

Care Me analyzed the data of EPB41L3, HPV16, and HPV18. It costs a third less than S5, and Care Me is undergoing clinical registration trials in China.

We created boxplots to illustrate the distribution of the S5 classifier histopathological diagnosis of the lesions (no lesion, LSIL, HSIL, and cervical cancer (CC)). We used the Mann-Whitney *U* test for comparing S5 scoring differences between different categories and the Cuzick test for the trend to determine if methylation increases significantly as a function of great histopathology results.

To maximize the sensitivity and specificity of the S5 score after the triage test (HPV16/18 and cytology), we created receiver operating characteristic (ROC) curve to estimate areas under the curve (AUC), and we obtained a new S5 cutoff value to discriminate ≥ (His)HSIL+ cases from < (His)HSIL+ cases. A second analysis and a third analysis were performed using the establishment of a 3.7 cutoff described in a Mexico triaged population and 0.8 cutoff described in the UK screening population for the detection of CIN2+ and CIN3+.

Based on the new S5 cutoff, we estimated sensitivity, specificity, positive predictive value (PPV), and negative predictive value (NPV) as the disease endpoint of HSIL+.

A ROC curve was also computed for comparing the performance of S5 and other existing methylation marker panels (“Care Me” and EPB41L3).

We followed up women with < (His)HSIL+ 1 year after colposcopy and calculated the regression rate in different S5 scoring subgroups.

All *p* values were estimated as two sided, with a confidence interval of 95%. Statistical analyses were performed by the SPSS statistical software version 25.0 (IBM, NY) and Excel. *p* < 0.05 was considered statistically significant.

## Data Availability

The full datasets are not publicly available due to the need to protect participant confidentiality; however, the data that support the findings of this study are available on request from the corresponding author. Inquiries should be communicated to corresponding author who will consider all sufficiently specified and reasonable requests.

## Abbreviations

ASCUS: Atypical squamous cells of undetermined significance
ASC-H: Atypical squamous cells, cannot exclude a high-grade squamous intraepithelial lesion
NILM: No intraepithelial lesion or malignancy
ROC: Receiver operating characteristic
SIL: Squamous intraepithelial lesion
CPG: Cytosine-phosphate-guanine site
hrHPV: High-risk human papillomavirus
No lesion: Histologically negative without squamous intraepithelial lesion
LSIL: Low-grade squamous intraepithelial lesion
HSIL: High-grade squamous intraepithelial lesion
CC: Cervical cancer
(His)HSIL: High-grade squamous intraepithelial lesion for histological diagnosis
(TCT)HSIL+: Cytological high-grade squamous intraepithelial lesion or worse for TCT result
L1: Late region 1
L2: Late region 2
LMIC: low- and middle-income country
PPV: Positive predictive value
NPV: negative predictive value

## References

[1] Tsikouras P, Zervoudis S, Manav B, Tomara E, Iatrakis G, Romanidis C, et al. Cervical cancer: screening, diagnosis and staging. Journal of BUON. 2016:21(2). 27273940

[2] et al. B Passamonti, D Gustinucci, P Giorgi Rossi, E Cesarini, S Bulletti, A Carlani, Cervical human papilloma virus (HPV) DNA primary screening test: results of a population-based screening programme in central Italy. J Med Screen. 2017:24(3). 10.1177/0969141316663580 27614992

[3] Torre LA, Bray F, Siegel R, Ferlay J, Lortet-Tieulent J, Jemal A. Global cancer statistics, 2012. CA: A Cancer Journal for Clinicians. 2012:65(2). 10.3322/caac.21262 25651787

[4] Clarke M, Gradissimo A, Schiffman M, Lam J, Sollecito C, Fetterman B, et al. Human papillomavirus DNA methylation as a biomarker for cervical precancer: consistency across 12 genotypes and potential impact on management of HPV-positive women. Clin Cancer Res. 2018:24(9). 10.1158/1078-0432.CCR-17-3251 PMC5932258 29420222

[5] Ahmad A, Raish M, Shahid M, Batra S, Batra V, Husain S. The synergic effect of HPV infection and epigenetic anomaly of the p16 gene in the development of cervical cancer. Cancer Biomark. 2017;19(4). 10.3233/CBM-160060 PMC13020740 28453456

[6] Kocsis A, Takacs T, Jeney C, Schaff Z, Koiss R, Jaray B, et al. Performance of a new HPV and biomarker assay in the management of hrHPV positive women: subanalysis of the ongoing multicenter TRACE clinical trial (n > 6,000) to evaluate POU4F3 methylation as a potential biomarker of cervical precancer and cancer. Clin Chem Lab Med. 2017;50(10). 10.1002/ijc.30534 27874187

[7] Pefoyo AK, Wang L, Gao J, Kupets R. Are women who exit colposcopy without treatment at elevated risk for cervical cancer? J Low Genit Tract Dis. 2017;21(1). 10.1097/LGT.0000000000000265 27749507

[8] Leeman A, Ebisch R, Kasius A, Bosgraaf R, Jenkins D, Van De Sandt M, et al. Defining hrHPV genotypes in cervical intraepithelial neoplasia by laser capture microdissection supports reflex triage of self-samples using HPV16/18 and FAM19A4/miR124-2 methylation. Gynecol Oncol. 2018;151(2). 10.1016/j.ygyno.2018.09.006 30219239

[9] L Torre, R Siegel, E Ward, A Jemal, Global cancer incidence and mortality rates and trends--an update. Cancer Epidemiol Biomarkers Prev. 2016:25(1). 10.1158/1055-9965.EPI-15-0578 26667886

[10] F Vink, Cjlm Meijer, G Clifford, M Poljak, A Ostrbenk, K Petry *et al*: FAM19A4/miR124-2 methylation in invasive cervical cancer: a retrospective cross-sectional worldwide study. International Journal of Cancer 2019, Aug 7. 10.1002/ijc.32614 PMC7383900 31390052

[11] Wentzensen N, Sun C, Ghosh A, Kinney W, Mirabello L, Wacholder S, et al. Methylation of HPV18, HPV31, and HPV45 genomes and cervical intraepithelial neoplasia grade 3. Journal of the National Cancer Institute. 2012;204(22). 10.1093/jnci/djs425 PMC3571257 23093560

[12] S Wilting, Ra Van Boerdonk, Fe Henken, Cj Meijer, B Diosdado, Ga Meijer *et al*: Methylation-mediated silencing and tumour suppressive function of hsa-miR-124 in cervical cancer. Molecular Cancer 2010, 2010 Jun 26;9. 10.1186/1476-4598-9-167 PMC2917428 20579385

[13] Fang J, Zhang H, Jin S. Epigenetics and cervical cancer: from pathogenesis to therapy. Tumour Biol. 2014:35(6). 10.1007/s13277-014-1737-z 24554414

[14] Heichman K, Warren J. DNA methylation biomarkers and their utility for solid cancer diagnostics. Clinical chemistry and laboratory medicine. 2012;50(10). 10.1515/cclm-2011-0935 23089699

[15] Lorincz A, Brentnall A, Vasiljevic N, Scibior-Bentkowska D, Castanon A, Fiander A, et al. HPV16 L1 and L2 DNA methylation predicts high-grade cervical intraepithelial neoplasia in women with mildly abnormal cervical cytology. International Journal of Cancer. 2013:1333. 10.1002/ijc.28050 PMC3708123 23335178

[16] De Strooper L, Berkhof J, Steenbergen R, Lissenberg-Witte BI, Snijders P, Meijer C, et al. Cervical cancer risk in HPV-positive women after a negative FAM19A4/mir124-2 methylation test: a post hoc analysis in the POBASCAM trial with 14 year follow-up. International Journal of Cancer. 2018:1436. 10.1002/ijc.31539 PMC6099282 29663363

[17] De Strooper L, Verhoef V, Berkhof J, Hesselink A, De Bruin H, Van Kemenade F, et al. Validation of the FAM19A4/mir124-2 DNA methylation test for both lavage- and brush-based self-samples to detect cervical (pre)cancer in HPV-positive women. Gynecol Oncol. 2016:141(2). 10.1016/j.ygyno.2016.02.012 PMC4851217 26921784

[18] Hernandez-Lopez R, Lorincz A, Torres-Ibarra L, Reuter C, Scibior-Bentkowska D, Warman R, et al. Methylation estimates the risk of precancer in HPV-infected women with discrepant results between cytology and HPV16/18 genotyping. Clinical Epigenetics. 2019:111. 10.1186/s13148-019-0743-9 PMC6790057 31606044

[19] Ar B, Vasiljevic N, Scibior-Bentkowska D, Cadman L, Austin J, Cuzick J, et al. HPV33 DNA methylation measurement improves cervical pre-cancer risk estimation of an HPV16, HPV18, HPV31 and {EPB41L3} methylation classifier. Cancer Biomark. 2015;155. 10.3233/CBM-150507 PMC12965437 26406956

[20] Cook D, Krajden M, Brentnall A, Gondara L, Chan T, Law JH, et al. Evaluation of a validated methylation triage signature for human papillomavirus positive women in the HPV FOCAL cervical cancer screening trial. International Journal of Cancer. 2019;144(10). 10.1002/ijc.31976 PMC6492122 30412281

[21] Lorincz A, Brentnall A, Scibior-Bentkowska D, Reuter C, Banwait R, Cadman L, et al. Validation of a DNA methylation HPV triage classifier in a screening sample. Int J Cancer. 2016:13811. 10.1002/ijc.30008 PMC4832297 26790008

[22] Hernandez-Lopez R, Lorincz ATA-OHOO, Torres-Ibarra L, Reuter C, Scibior-Bentkowska D, Warman R, et al. Methylation estimates the risk of precancer in HPV-infected women with discrepant results between cytology and HPV16/18 genotyping. Clin Epigenetics. 2019:111. 10.1186/s13148-019-0743-9 PMC6790057 31606044

[23] Chen Y, Rl H, Yk H, Liao Y, Ph S, Wang H, et al. Methylomics analysis identifies epigenetically silenced genes and implies an activation of beta-catenin signaling in cervical cancer. International Journal of Cancer. 2014:1351. 10.1002/ijc.28658 24310984

[24] Lai HC, Ou YC, Chen TC, Huang HJ, Cheng YM, Chen CH, Chu TY, Hsu ST, Liu CB, Hung YC, et al. PAX1/SOX1 DNA methylation and cervical neoplasia detection: a Taiwanese Gynecologic Oncology Group (TGOG) study. Cancer Medicine. 2014;34. 10.1002/cam4.253 PMC4303175 24799352

[25] Pun PB, Liao YP, Su PH, Wang HC, Chen YC, Hsu YW, et al. Triage of high-risk human papillomavirus-positive women by methylated POU4F3. Clinical Epigenetics. 2015;217. 10.1186/s13148-015-0122-0 PMC4546171 26300990

[26] Hesselink AT, Heideman DA, Steenbergen RD, Coupé VM, Overmeer RM, Rijkaart D, Berkhof J, et al. Combined promoter methylation analysis of CADM1 and MAL: an objective triage tool for high-risk human papillomavirus DNA-positive women. Clinical Cancer Research. 2011:1517. 10.1158/1078-0432.CCR-10-2548 21389098

[27] Eijsink JJ, Lendvai Á, Deregowski V, Klip HG, Verpooten G, Dehaspe L, de Bock GH, et al. A four-gene methylation marker panel as triage test in high-risk human papillomavirus positive patients. International Journal of Cancer. 2012:15130. 10.1002/ijc.26326 21796628

[28] Hansel A, Steinbach D, Greinke C, Schmitz M, Eiselt J, Scheungraber C, et al. A promising DNA methylation signature for the triage of high-risk human papillomavirus DNA-positive women. PLoS One. 2014;199. 10.1371/journal.pone.0091905 PMC3960142 24647315

[29] Hesselink AT, Heideman DA, Steenbergen RD, Gök M, van Kemenade FJ, Wilting SM, Berkhof J, et al. Methylation marker analysis of self-sampled cervico-vaginal lavage specimens to triage high-risk HPV-positive women for colposcopy. International Journal of Cancer. 2014:15135. 10.1002/ijc.28723 24474183

[30] Schmitz MA-OHOO, Eichelkraut K, Schmidt D, Zeiser I, Hilal Z, Tettenborn Z, et al. Performance of a DNA methylation marker panel using liquid-based cervical scrapes to detect cervical cancer and its precancerous stages. BMC Cancer. 2018:181. 10.1186/s12885-018-5125-8 PMC6276155 30509219

[31] Ma C, Luhn P, Gage J, Bodelon C, Dunn S, Walker J. Discovery and validation of candidate host DNA methylation markers for detection of cervical precancer and cancer. International Journal of Cancer. *et al*, 2017;141(4):4. 10.1002/ijc.30781 PMC6774256 28500655

[32] Ortoft G, Henriksen T, Hansen E, Petersen L. After conisation of the cervix, the perinatal mortality as a result of preterm delivery increases in subsequent pregnancy. BJOG. 2010;117(3). 10.1111/j.1471-0528.2009.02438.x 19943823

[33] K Van Velthoven, W Poppe, H Verschuere, M Arbyn: Pregnancy outcome after cervical conisation: a 2nd retrospective cohort study in the Leuven University Hospital. Eur J Obstet Gynecol Reprod Biol 2017, 2017 sep;216. 10.1016/j.ejogrb.2017.06.043 28822944

[34] Wentzensen N, Schiffman M, Mi S, Mj K, Rb P, Km S, et al. ASCCP colposcopy standards: risk-based colposcopy practice. J Low Genit Tract Dis. 2017:214. 10.1097/LGT.0000000000000334 28953111

[35] Y Tian, W Yuan, Y Liou, C Yeh, L Cao, Y Kang *et al*: Utility of gene methylation analysis, cytological examination, and HPV-16/18 genotyping in triage of high-risk human papilloma virus-positive women. Oncotarget 2018, 8(37). 10.18632/oncotarget.19459 PMC5617504 28977944

